# Supplementary material for: Ordovician–Silurian true polar wander as a mechanism for severe glaciation and mass extinction
Source: Nat Commun. 2022 Dec 26;13:7941. doi: 10.1038/s41467-022-35609-3 (PMC9792554; doi:10.1038/s41467-022-35609-3)
Supplement: Supplementary file 1 — Supplementary Information [file 41467_2022_35609_MOESM1_ESM.pdf]

Supplementary Information for

Ordovician–Silurian true polar wander as a mechanism for severe glaciation and mass extinction

Xianqing Jing[1], Zhenyu Yang[1*], Ross N. Mitchell[2*], Yabo Tong[3], Min Zhu[4], Bo Wan[2]

[1]College of Resources, Environment and Tourism, Capital Normal University, Beijing, China.

[2]State Key Laboratory of Lithospheric Evolution, Institute of Geology and Geophysics, Chinese Academy of Sciences, Beijing, China.

[3]Institute of Geomechanics, Chinese Academy of Geological Sciences, Beijing, China. [4]Key Laboratory of Vertebrate Evolution and Human Origins of Chinese Academy of Sciences, Institute of Vertebrate Paleontology and Paleoanthropology, Chinese Academy of Sciences, Beijing, China.

Correspondence to: Z. Y. Yang (zhenyu.yang@cnu.edu.cn)

Ross N. Mitchell (ross.mitchell@mail.iggcas.ac.cn),

This PDF file includes:

Supplementary text

Supplementary Figs. 1-7

Supplementary Tables. 1-4

Supplementary references

Palaeomagnetism and rock magnetism

The remanence directions were analyzed using least-squares analysis[1] or great circle intersections[2] as employed by the Paleomagnetism.org web application[3]. Site-mean directions were calculated by averaging the different samples, and the statistical parameters were calculated assuming a Fisher distribution[4]. Backfield demagnetization curves unmixing, or coercivity spectra, was analyzed by using the Max Unmix web application[5]. PaleoMac[6] was used to conduct the fold and reversal test and produce Fig. 1 and Supplementary Figs. 3, 4, 5. Pmagpy[7] was used to calculate the great circle, palaeolatitude of different time and the virtual geographic poles from site-mean remanence directions.

Two magnetic components were revealed (Fig. 1, Supplementary Figs. 3, 4). A low-temperature component, which was isolated below 300 ºC, resembles the present-local geomagnetic field (Supplementary Fig. 7). Hence, it is interpreted as most likely representing a recent viscous remanent magnetization (VRM). Thereafter, a high-temperature component was separated with unblocking temperatures ranging from 620–670 ºC , mostly higher than 650 ºC (Fig. 1, Supplementary Figs. 3, 4), suggestive of a chemical remanent magnetization (CRM) and/or a post-depositional remanent magnetization (pDRM) carried by hematite.

Statistical analysis reveals that the site-mean directions from the sections Kapeng (SK) and Tianlu (ST) are distinct from those of the section Yongdong (SY, Supplementary Fig. 5a, b, Supplementary Table 1). The virtual geomagnetic poles (VGPs) calculated from the sections SK and ST data resemble the known Triassic results of South China (Supplementary Fig. 5c), which suggests remagnetization during this period. These results confirm the observation of widespread early Mesozoic remagnetization in South China[8]. This comparison also argues that the distinct direction of the SY section has not suffered younger remagnetization.

Results of the SY section recorded both east-and-down and a west-and-up vectors (Figs. 1a, d, g, 2, Supplementary Fig. 3, Supplementary Table 1). With only one

reversed polarity site, the SY section is precluded from a reversal test. As all the SY section sites come from the west limb, we cannot conduct a fold test, but a regional fold test is attempted next based on comparison with coeval data from other sections.

The K value of dispersion for the mean virtual geomagnetic pole (VGP) of the SY sites is 90.3 (Supplementary Table 1), which is >70 and suggests it may not average out the palaeosecular variation (PSV)[9]. We do note that if the sedimentary data are treated at the sample level instead of the site level, the K value for 101 samples is 65.5, where both these values satisfy the requirements of Reliability criterion #2 of Meert et al.[9] concerning the averaging of PSV. Nonetheless, the A95 (1.81) resulting from the sample-level mean VGP is slightly below the A95min (1.95) of Deenen et al.[10], again suggesting that PSV may be slightly under-sampled by the SY section. We next sought to combine our results from the SY section with previous results from coeval strata in order to adequately average PSV.

Previous studies[11,12] mixed results from the Rongxi Formation (Fm), the Huixingshao Fm, and the Xiaoxi Fm of different regions together, which reduces the temporal resolution of the Silurian palaeopoles. Therefore, we reanalyze those paleomagnetic data of the Rongxi Fm of Huang et al.[11] and Opdyke et al.[12] (Supplementary Table 1). After reviewing the papers of Huang et al.[11] and Opdyke et al.[12], we note that data from the Rongxi section, Xiushan county of Opdyke et al.[12] are both from the Rongxi and Huixingshao formations (Supplementary Fig. 1), which cannot be separated from each other. The K value of VGPs of these 12 sites is 121.6 (Supplementary Table 1), which suggests they did not average out the secular variation either.

The two Guandi Fm data from Qujing (Supplementary Fig. 1) were thought to be contemporary with the Huixingshao Fm[12]. However, new palaeontologic studies suggest that the Huixingshao Fm belongs to the middle Telychian and the Guandi Fm covers the Ludlow Epoch[13,14]. Specifically, the red beds of the Guandi Fm (Guandi II and Guandi III Members) are of late Gorstian to early Ludfordian Age[14] (Supplementary Fig.1). Therefore, these two data have a distinct age from the data from the Rongxi and Huixingshao formations, hence we reject them for our combined

calculation.

Besides that, data of sites 6 and 7 from the Shiqian section are from the Huixingshao Fm (Supplementary Fig. 1). The remaining data including sites 8–14 from the Shiqian section[11] and site A from the Songkan section, Tongzi[12], both of which clearly belong to the Rongxi Fm. The K value of these 8 sites is 133.7 (Supplementary Table 1), which still exceeds 70. Even if we combined all the data from the Rongxi and Huixingshao formations of Huang et al.[11] and Opdyke et al.[12], comprising 22 sites, the K value of 87.6 still exceeds 70 (Supplementary Table 1). These results suggest that all the previous data cannot average PSV, and hence are unreliable on their own. Nonetheless, considering that the Rongxi and Huixingshao formations span only ~4 Myr[13,15,16], we combine our Huixingshao Fm data with all these data from the Rongxi and Huixingshao formations of Huang et al.[11] and Opdyke et al.[12], totaling 28 sites. The mean of the combined VGPs has a K = 48.4 (Supplementary Table 1), which is squarely in the acceptable range of 10–70 and suggests PSV is adequately averaged in the new combined result. These data also pass a fold test[17] ("Xi1" test) at 99% confidence ($Xi1 = 5.956 < Xi1_{critical} = 8.703$) . The mean pole of these VGPs (S_1M) is located at 6.8ºN, 195.6ºE, with A95 = 4 (Fig. 1f). This S_1M pole fulfills 6 of 7 reliability criteria proposed by Meert et al.[9], and hence is a very reliable palaeomagnetic pole.

Coercivity spectra demonstrated that all specimens contain two coercivity components, one low coercivity (~270 mT) and one high coercivity (660–700 mT) (Supplementary Fig. 6). The κ-T curve from the ST section demonstrates an irreversible characteristic and its susceptibility increased dramatically around 550–580°C during cooling, suggesting mineral transformation to magnetite (Supplementary Fig. 6). During heating, the susceptibility only decreased after 600°C and until 700°C. The κ-T curve of the SY section specimen is different from the ST section. It decreased dramatically after 600°C until 660°C, and then remained stable (Supplementary Fig. 6), without evidence of a mineral transformation during heating. These observations collectively indicate that our samples only contain hematite as a main magnetic carrier. But samples from the ST section have more complex combinations with hematite and mineral transformation. Meanwhile, an interpretation of hematite in the SY section is

straightforward. Considering these collective thermal demagnetization and rock magnetism results, we argue that the result from the SY section is a primary record, however the results from both the SK and ST sections should represent an early Mesozoic remagnetization.

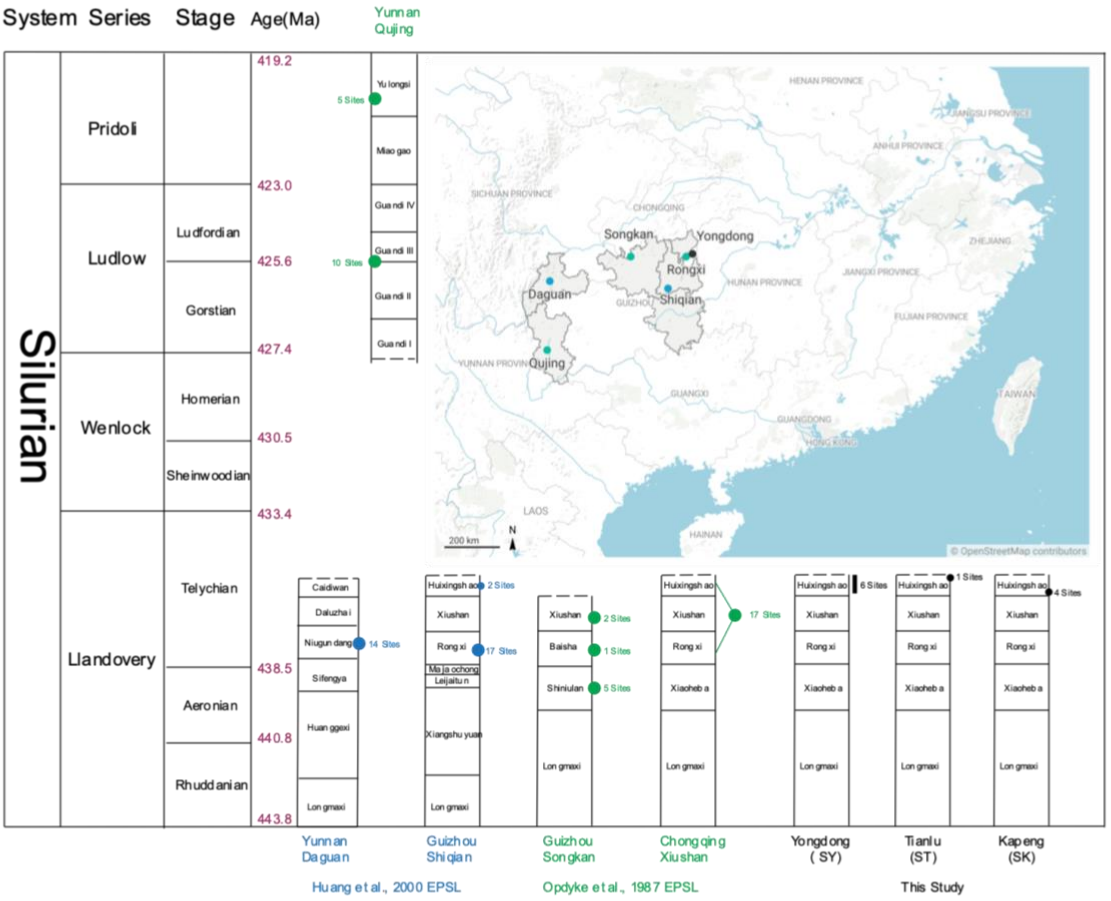

Supplementary Fig. 1. Sampling horizons and locations of our sections and those of previous studies. Opdyke et al.[12] (green) and Huang et al.[11] (blue) sampled the Rongxi Formation (Fm) intensively. Our samples are all from the Huixingshao Fm (black). Map was generated by Datawrapper website (https://www.datawrapper.de/). Map data from OpenStreetMap.

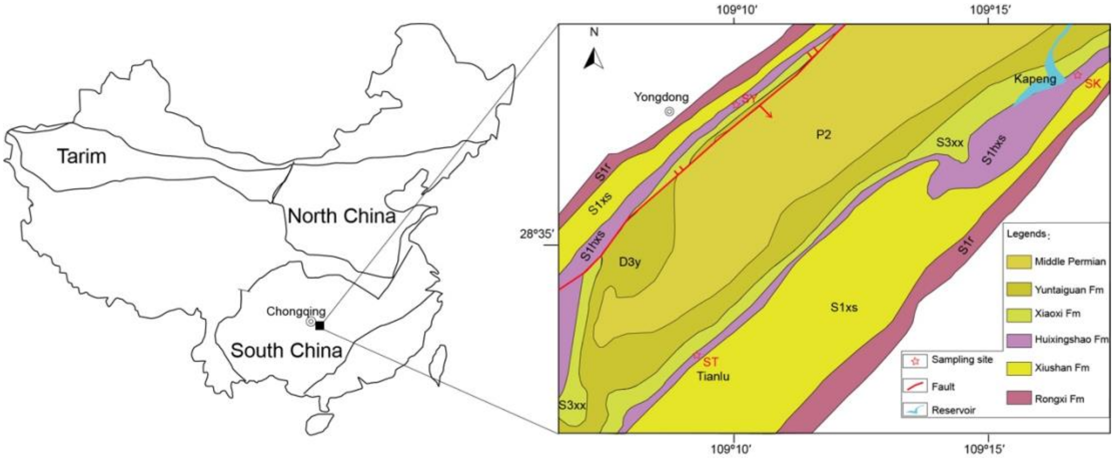

Supplementary Fig. 2. Sampling location and the geologic setting of the sampled sections. Sampling sections are in Xiushan County, Chongqing, China. Three sections are close to Yongdong village (SY), Kapeng reservoir (SK), and Tianlu road (ST, near Chuanhegai village). Geologic setting map is modified from the Extended data Fig. 1 of refence[18].

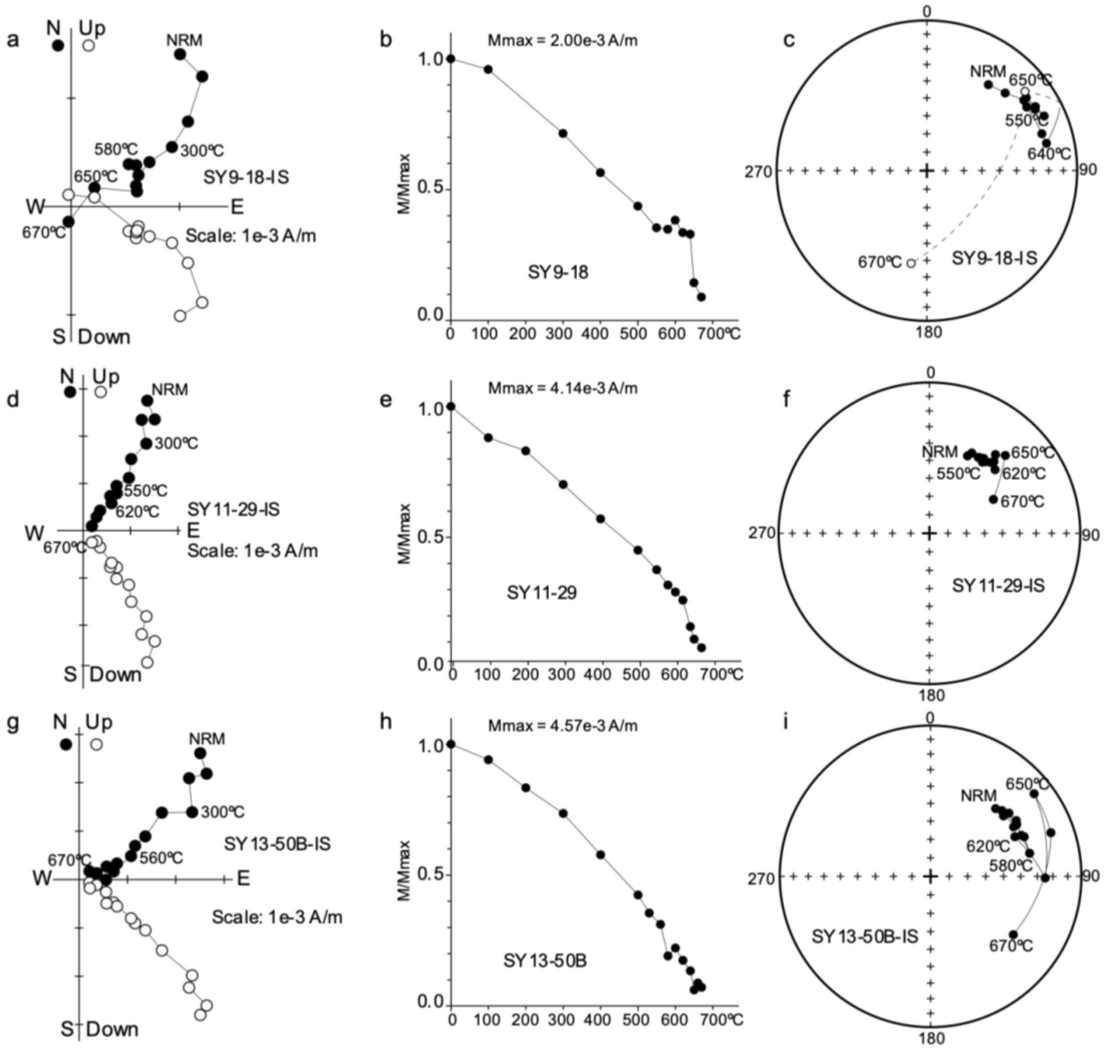

Supplementary Fig. 3. Demagnetization behaviour of the section in Yongdong (SY). Zijderveld plots (**a, d, g**), normalized stepwise thermal decay curves (**b, e, h**) and equal area projections (**c, f, i**) of the thermal demagnetization of representative samples from the section Yongdong (SY) in geographic coordinates. In the Zijderveld plots, black and white dots represent horizontal and vertical projections, respectively. While, in the equal area projections, they represent directions plotted in the lower and upper hemispheres, respectively. All figures were generated by PaleoMac[6].

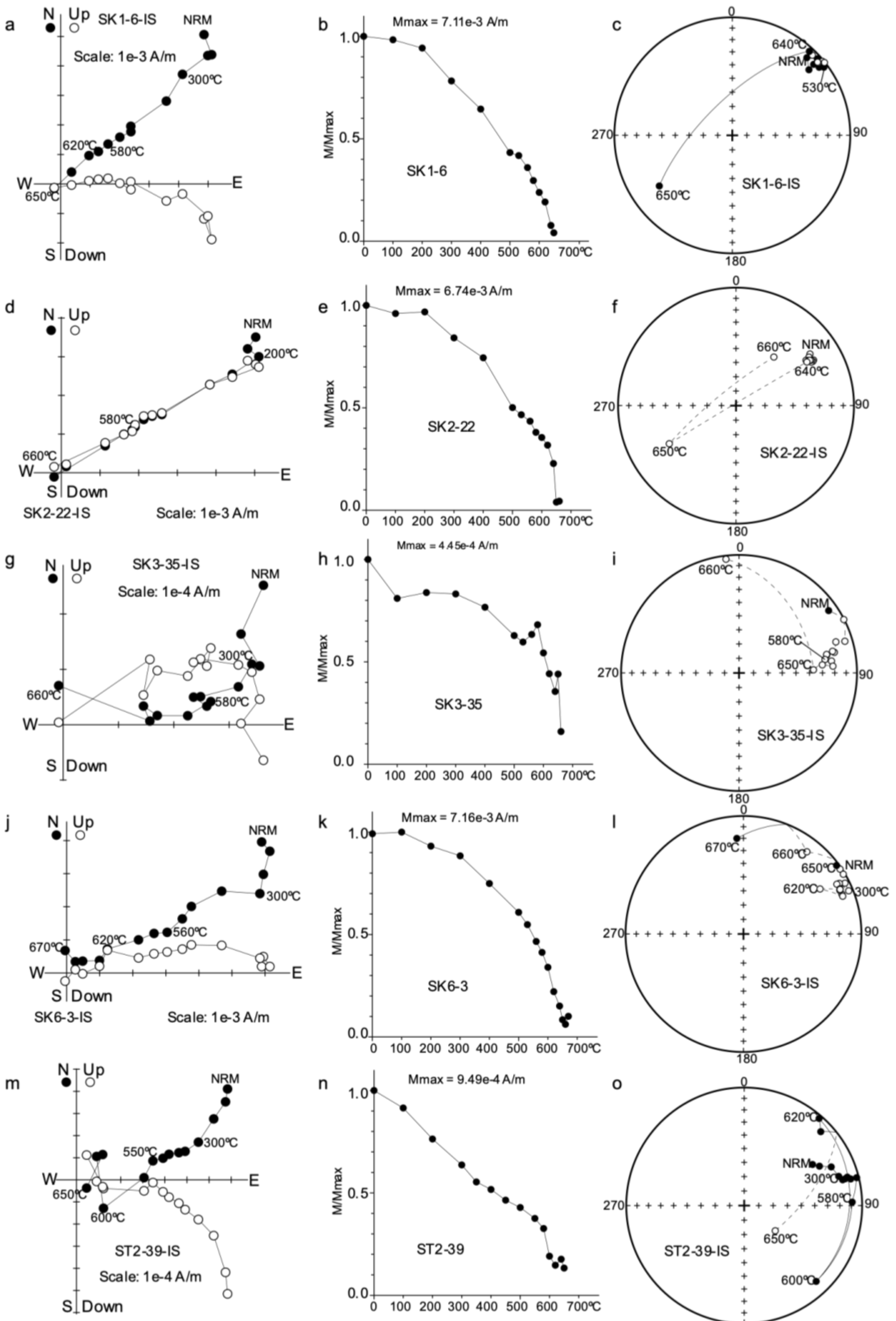

Supplementary Fig. 4. Demagnetization behaviour of the sections in Kapeng (SK) and Tianlu (ST). Zijderveld plots (**a, d, g, j, m**), normalized stepwise thermal decay curves (**b, e, h, k, n**) and equal area projections (**c, f, i, l, o**) of the thermal

demagnetization of representative samples from the sections Kapeng (SK) and Tianlu (ST) in geographic coordinates. In the Zijderveld plots, black and white dots represent horizontal and vertical projections, respectively. While, in the equal area projections, they represent directions plotted in the lower and upper hemispheres, respectively. All figures were generated by PaleoMac[6]

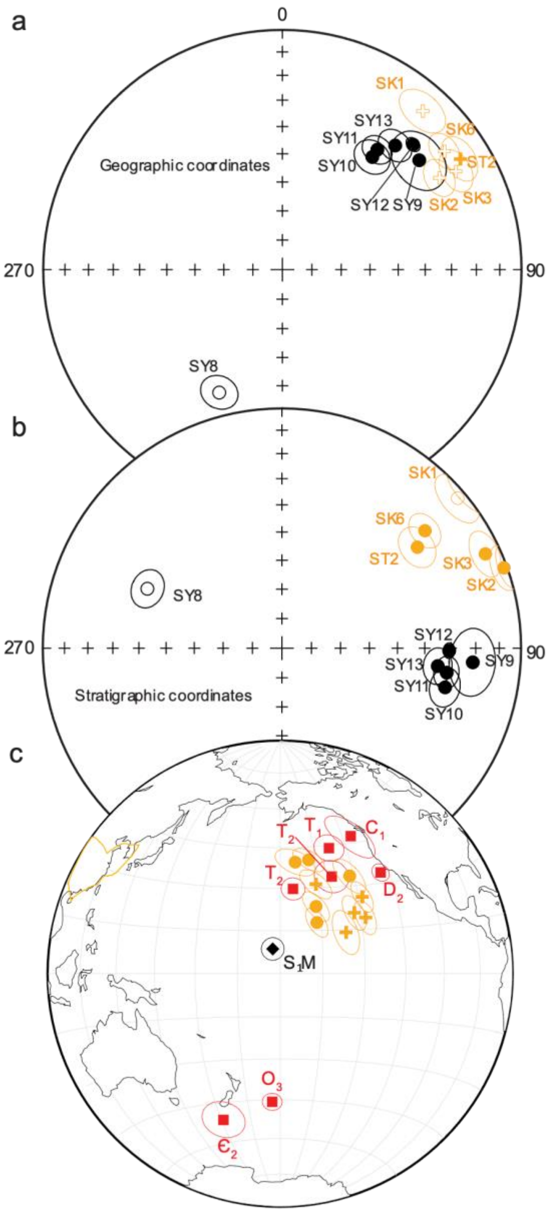

Supplementary Fig. 5. a, b Equal area stereographic projection of site mean directions of the high-temperature components of the Huixingshao Fm from this study in geographic and stratigraphic coordinates respectively. Virtual geomagnetic poles (VGPs) of of sites ST2 and SK1, 2, 3, 6 (yellow) are plotted in c. **c** Comparison of VGPs of sites ST2 and SK1, 2, 3, 6 (yellow) with the existing poles (red squares) of South China[19], which suggests that they may be a Triassic remagnetization. Crosses and dots are in geographic and stratigraphic coordinates respectively. All figures were generated by PaleoMac[6]

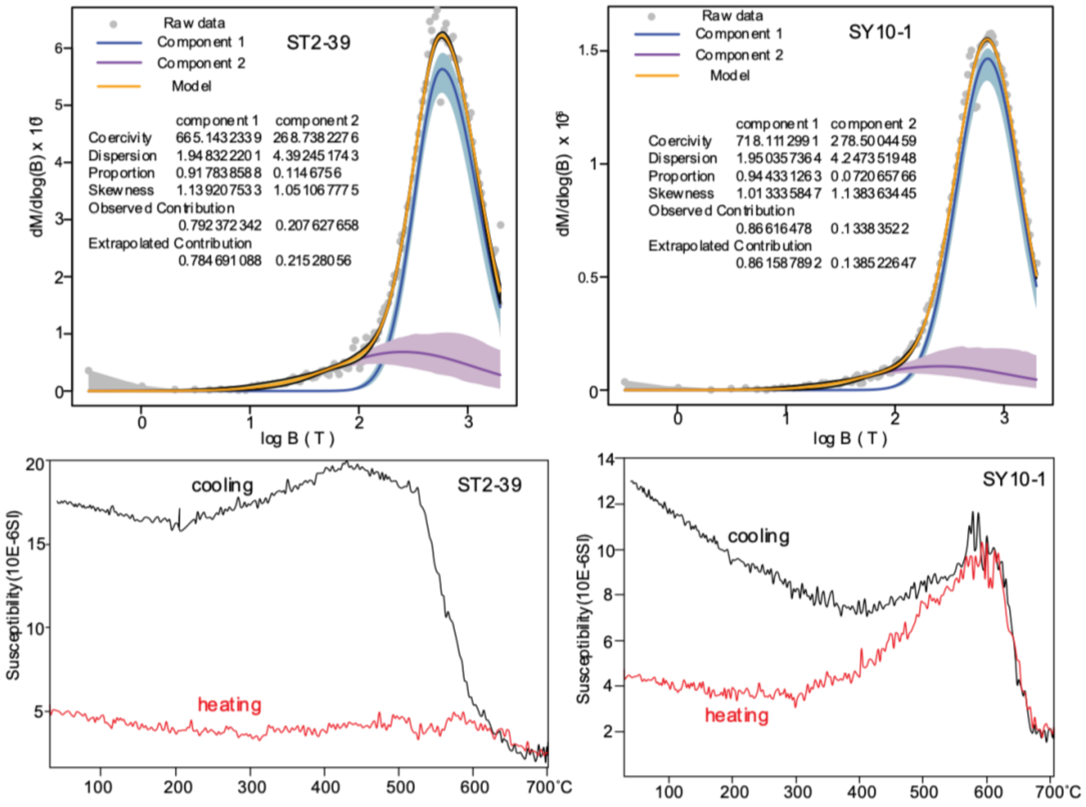

Supplementary Fig. 6. Rock magnetic experiments. (Top) Model fits for samples ST2-39 and SY10-1 using web application Max UnMix[5]. (Bottom) Thermal susceptibility curves of these two samples with heating and cooling between room temperature to ~700ºC.

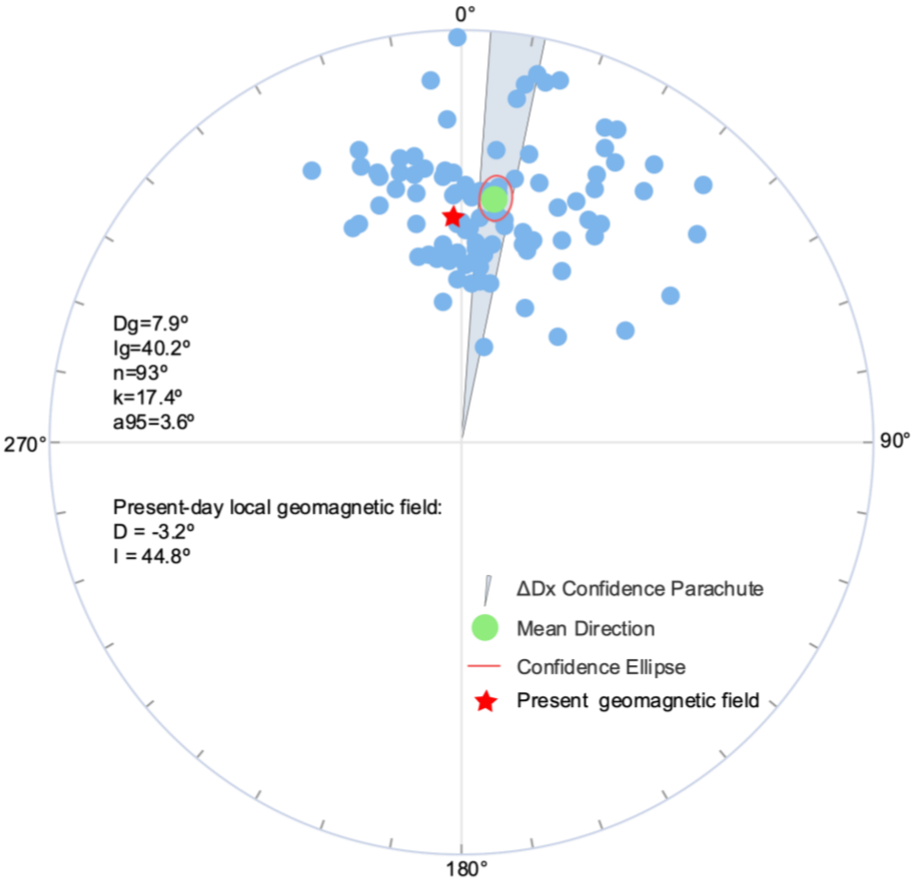

Supplementary Fig. 7. Equal area projection of low temperature component. Blue dots are the specimens' data from sections Yongdong (SY), Kapeng (SK) and Tianlu (ST). The mean direction is close to the local present day geomagnetic field. Drafted using the Paleomagnetism.org[3] website.

Supplementary Table 1. Data from high-temperature component of the Huixingshao Fm, Xiushan, Chongqing (109.163°E, 28.613°N) and previous data from Silurian strata of South China.

	Site	Comment	n/N	Dg	Ig	Ds	Is	ks	a95	Plat	Plong	Reference
Shiqian	6	HXS	5	77.7	-47.4	79.9	-2.6	154	9	8.3	203.9	Huang et al.[11]
	7	HXS	7	67.6	-21.3	67.5	22	65.6	7.5	25.1	197.8	
	8	RX	6	43.4	52.3	77.7	33.5	222.4	4.5	18.9	186.7	
	9	RX	7	63.6	39.7	80.2	14.9	125.4	5.4	12.2	195.7	
	10	RX	8	61.3	43.7	81.1	19.2	277	3.3	12.4	193.2	
	11	RX	8	57.6	36.1	74	14.7	269.8	3.4	17.6	198.7	
	12	RX	3	54.3	39.1	73.8	18.7	478.4	5.6	18.7	196.7	
	13	RX	6	54.5	41	75.2	20.1	88.5	7.2	17.8	195.4	
	14	RX	6	78.4	34.8	87.9	4.7	73.5	7.9	2.9	196.6	
	15	R	7	54.5	-18.3	64.5	-1.8	170.8	5.6	22	211.3	
	16	R	5	57.6	-17.2	65.4	1.2	50.3	15.9	22	209.4	
	17	R	7	59.6	-19.1	68	1.4	153.8	5.9	19.7	207.9	
	18	R	5	51.7	-16.7	60.6	-2.4	71.8	13.3	25.2	213.7	
	19	R	6	65.3	-27.4	76.8	-0.3	38.1	14.2	11.6	204.3	
Daguan	5	R	6	60.4	14.7	60.5	3.7	68.4	10.6	26.7	207	Huang et al.[11]
	6	R	5	58.6	15.9	58.8	5	55.7	15.1	28.5	207.3	
	8	R	6	74.5	2.5	74.6	-8.4	152	5.5	11.5	205	
	9	R	5	64	6	64	-7	68.1	9.3	21	210	
	10	R	6	61.3	15.1	61.2	2.1	83.5	7.4	25.7	207.3	
	11	R	6	72.3	16.3	65.3	2.2	48.8	9.7	22.2	205	
	12	R	5	70.8	13.7	65.9	-0.7	219.3	5.2	21	206	
	13	R	6	72.8	5.6	71.2	-1.4	86.2	7.3	16.2	203.6	
	14	R	6	66	15.6	59.6	1.9	230.4	4.4	27.1	208.3	

Songkan, Tongzi	Z	RX	5	20.1	59.9	70.1	20.1	84	8.4	22.3	197	Opdyke et al.[12]
Xiushan	J	RX+HXS	4	35.4	52.5	86.1	18.6	414.6	4.5	8	192.4	Opdyke et al.[12]
	K	RX+HXS	5	57.3	45.7	85.3	2.7	218.4	5.2	4.7	200	
	L	RX+HXS	4	57.1	40.2	86.1	11.2	294.7	5.4	6.1	195.8	
	M	RX+HXS	5	61.9	43.6	89.8	8.7	1433.1	2	2.3	195.2	
	N	RX+HXS	4	58.3	53.9	97.5	16.1	319.4	5.2	-2.6	188.1	
	O	RX+HXS	4	60.3	47	92.1	11.6	351.9	4.9	1	192.8	
	P	RX+HXS	5	67.7	46.9	88.2	13.7	219.2	5.2	4.8	193.7	
	X	RX+HXS	5	66.1	42.4	84.5	10.7	125.2	6.9	7.4	196.8	
	R	RX+HXS	5	59.6	41.9	80.3	13.4	193.4	5.5	11.7	197.5	
	S	RX+HXS	4	87.5	-8.3	82.1	31	57.2	12.3	14.6	187.6	
	T	RX+HXS	5	74.9	-21.6	80.1	0.7	1917.5	1.8	8.9	203.4	
	V	RX+HXS	3	68.1	-26.9	74.6	5.7	184.4	9.1	14.9	203.8	
Qujing	F	GD	5	282.4	8.7	283.8	-4.4	43.9	11.7	11.5	365.7	Opdyke et al.[12]
	G	GD	5	279.4	21.5	289.7	10.9	69.3	9.3	20.1	370.2	
Kapeng	SK1	R	17	41.5	-12.4	49.6	-3.9	25.3	7.2	33.5	223.2	This study
	SK2	R	10	59.7	-24.9	70.2	1.5	74	5.7	17.7	208.3	
	SK3	R	14	60.4	-17.5	65.2	7	41.5	6.2	23.4	208.4	
	SK6	R	12	53.9	-17.1	50.7	23.7	62.3	5.5	40.2	207.3	
Tianlu	ST2	R	10	58	13.5	53.4	30.1	55.7	6.5	39.5	201.4	
Yongdong	SY8	HXS	8	207.3	-41.9	293.8	-38.5	87.2	6	-8.8	168.5	
	SY9	HXS	8	51.4	27.7	94.3	21	34.9	9.5	1.5	187.6	
	SY11	HXS	19	38.4	36.3	98.5	31	48.5	4.9	0.8	180.5	
	SY12	HXS	41	46.3	25.4	90.9	30.7	96.6	2.3	7.1	184.2	
	SY13	HXS	8	42.3	30.6	96.6	34.7	102.2	5.5	3.5	179.3	

SY10	HXS	20	218.6	-39.6	283.5	-30.5	32	5.9	-3.5	178.3
Mean VGP of Huixingshao Fm data of this study									0.1	179.7
									K=90.3	A95=7.1
Mean VGP of Rongxi Fm data (Shiqian sites 8-14 and Tongzi site z)									15.4	195
									K=133.7	A95=4.8
Mean VGP of mixed Rongxi Fm and Huixingshao Fm data from Xiushan (12 sites)									6.8	195.6
									K=121.6	A95=3.9
Mean VGP of Rongxi Fm and Huixingshao Fm data of Huang et al. and Opdyke et al. (22 sites)									10.8	195.8
									K=87.6	A95=3.3
Site-mean direction and mean VGP of all data from Rongxi Fm and Huixingshao Fm (S_1M pole)										
		28	57.4	32.4	85.1	18	31.2	5	8.6	192.4
									K=48.4	A95=4.0

Note: *n*/N, number of samples/sites for statistical analysis; Dg, Ig, Ds, Is, declination and inclination in geographic and stratigraphic coordinates; k, Fisher precision parameter of the mean; α95, confidence of the mean direction; dp/dm, semi-axes of elliptical error around the pole at a probability of 95%; Plat/Plong, latitude and longitude of the palaeomagnetic pole; K, Fisher's precision parameter in pole space; A95, cone of confidence about the mean paleomagnetic pole. HXS, Huixingshao Formation (Fm); RX, Rongxi Fm; HXS+RX, cannot determine whether the data were from Rongxi Fm or Huixingshao Fm; GD, Guandi Fm; R, remagnetized data.

Supplementary Table 2. 460–430 Ma paleopoles used for paleogeographic reconstruction.

Continent	Age (Ma)	Latitude (˚N)	Longitude (˚E)	dp (˚)	dm (˚)	K	R/Rf	Type	References	Angle Distance/Angle Velocity (time gap = 10 Ma)
South China	450	45.8	11.3	2.4	4.2	66.2	7	PP	Han et al.[20], This Study, Huang et al.[11], Opdyke et al.[12]	54.4º/5.44º Ma^{-1}
	438-435	-8.6	12.4	4	4	48.4	6/6	PP		
Tarim	460	33.7	5	2.7	4	21.3	7	PP	Huang et al.[21]	54.0º/5.4º Ma^{-1}
	433	-17.7	350.6	6.9	6.9	44.3	7	PP	this study, Huang et al.[21]	
Siberia	463	23	338	4	4		6/4	PP	Gallet and Pavlov[22]	
	439	-3	298	13.1	13.1		5/2,4	PP	Torsvik et al.[23]	
Baltica	460	17.9	47.5	7.2	8.5			SP	Wu et al.[24]	55.0º/5.5º Ma^{-1}
	450	13.2	41.9	10	16.2			SP		
	440	-12.6	352.9	10.9	14.2			SP		
	430	-10.3	345.8	9	10			SP		
Laurentia	460	-12.9	314.6	11	16.9			SP	Wu et al.[24]	
	450	-10.6	304.3	12.7	17.8			SP		
	440	-12.3	304.9	11.6	15.2			SP		
	430	-15.1	295.2	8.3	9.6			SP		
Gondwana	460	16.9	17.1	14.6	15.4			SP	Wu et al.[24]	58.4º/5.84º Ma^{-1}
	450	14.9	17	2.4	2.4			SP		
	440	-43.3	12	18.5	18.6			SP		
	430	-47.4	13	15.1	17.1			SP		

Note: R, reliability criteria of palaeomagnetic data including 7 aspects: 1. age constrained to within ± 15 Ma; 2. Stepwise demagnetization effectiveness confirmed by multiple demagnetization methods. Test for averaging of PSV. $N \geq 25$, $10 \leq K \leq 70$, $B \geq 8$ sites (minimum 3 samples/site); 3. Rock magnetic and/or microscopic examination and identification of magnetic carriers; 4. Fold/tilt test, baked contact tests, conglomerate test or other field tests that constrain age of magnetization; 5. Data from thrust sheets or intrusive rocks must be younger than the last tectonic deformation in the area. Detrital sedimentary rocks that do not require inclination corrections will meet this criterion; 6. Statistically significant antipodal normal and reverse directions Ra, Rb or Rc rated or show support for a common mean; 7. No resemblance to younger poles by more than a period based on overlapping A95. Rf, failed R criterion of the data. K, Fisher's precision parameter in pole space. PP- Palaeomagnetic pole; SP- Synthetic poles from averages.

Supplementary Table 3. 460–430 Ma paleopoles from Torsvik et al. for comparison.

Continent	Age (Ma)	Latitude (˚N)	Longitude (˚E)	A95 (˚)	Type	References
Baltica	460	9.1	39.7	7.2	SP	Torsvik et al.[25]
	450	4	34.5	4.9	SP	
	440	-8.7	12.2		SP	
	430	-20.1	348.5	3.3	SP	
Laurentia	460	-18.3	329.5		SP	
	450	-21.1	328.1		SP	
	440	-24	326.6	7.3	SP	
	430	-20.3	313.1	17.5	SP	
Gondwana	460	32.7	351.5		SP	
	450	25	343	18	SP	
	440	25	343	18	SP	
	430	1.9	351.6		SP	

Note: SP- Synthetic poles from averages

Supplementary Table 4. Euler rotation parameters for the continents in our reconstruction.

Rotated Continent	Age (Ma)	Euler pole		Euler angle (°)	Fixed Continent
		Latitude	Longitude		
Siberia	440–460	46.7	11.4	73.4	North West Africa
Baltica	440–460	-7.9	21.3	55.6	North West Africa
Laurentia	440	27.3	358.8	69.8	North West Africa
Laurentia	445	30.8	339.7	85.1	North West Africa
Laurentia	460-450	31.5	325.4	104.5	North West Africa
South China	440–460	-1.3	129.4	30.7	North West Africa
Lhasa	440–460	3.2	355.4	-40.6	North West Africa
Sibumasu	440–460	14	81.1	-83.6	North West Africa
Tarim	440–460	35.2	74.6	31.2	North West Africa
Australia	440–460	-28.1	293.2	52.1	North West Africa
Arabia	440–460	26.2	11.2	-14.2	North West Africa
Madagascar	440–460	-14.9	277.6	15.7	North West Africa
India	440–460	26.7	37.3	-69.4	North West Africa
South Qiangtang	440–460	30.2	45.5	-63	North West Africa
East Antarctica	440–460	-12.4	326.2	53.3	North West Africa
South America	440–460	53	325	51	North West Africa

Note: Positive Euler angles denote counterclockwise rotations.

Supplementary References:

1. Kirschvink, J. L. The least-squares line and plane and the analysis of palaeomagnetic data. *Geophys. J. Int.* **62**, 699–718 (1980).
2. McFadden, P. L. & McElhinny, M. W. The combined analysis of remagnetization circles and direct observations in palaeomagnetism. *Earth Planet. Sci. Lett.* **87**, 161–172 (1988).
3. Koymans, M. R., Langereis, C. G., Pastor-Galán, D. & van Hinsbergen, D. J. J. Paleomagnetism.org: An online multi-platform open source environment for paleomagnetic data analysis. *Comput. Geosci.* **93**, 127–137 (2016).
4. Fisher, R. Dispersion on a Sphere. *Proc. R. Soc. Math. Phys. Eng. Sci.* **217**, 295–305 (1953).
5. Maxbauer, D. P., Feinberg, J. M. & Fox, D. L. MAX UnMix: A web application for unmixing magnetic coercivity distributions. *Comput. Geosci.* **95**, 140–145 (2016).
6. Cogné, J. P. PaleoMac: A Macintosh™ application for treating paleomagnetic data and making plate reconstructions. *Geochem. Geophys. Geosystems* **4**, (2003).
7. Tauxe, L. *et al.* PmagPy: Software package for paleomagnetic data analysis and a bridge to the Magnetics Information Consortium (MagIC) Database. *Geochem. Geophys. Geosystems* **17**, 2450–2463 (2016).
8. Jiao, W., Li, Y.-X., Yang, Z. & Liu, J. A widespread Early Mesozoic remagnetization in South China. *J. Geophys. Res. Solid Earth* **124**, 88–103 (2019).
9. Meert, J. G. *et al.* The magnificent seven: A proposal for modest revision of the quality index. *Tectonophysics* **790**, 228549 (2020).
10. Deenen, M. H. L., Langereis, C. G., van Hinsbergen, D. J. J. & Biggin, A. J. Geomagnetic secular variation and the statistics of palaeomagnetic directions: Statistics of

palaeomagnetic directions. *Geophys. J. Int.* **186**, 509–520 (2011).

11. Huang, K., Opdyke, N. D. & Zhu, R. Further paleomagnetic results from the Silurian of the Yangtze Block and their implications. *Earth Planet. Sci. Lett.* **175**, 191–202 (2000).
12. Opdyke, N. D., Huang, K., Xu, G., Zhang, W. Y. & Kent, D. V. Paleomagnetic results from the Silurian of the Yangtze paraplatform. *Tectonophysics* **139**, 123–132 (1987).
13. Chen, Z. *et al.* Age of the Silurian Lower Red Beds in South China: Stratigraphical Evidence from the Sanbaiti Section. *J. Earth Sci.* **32**, 524–533 (2021).
14. Cai, J., Zhao, W. & Zhu, M. Subdivision and age of the Silurian fish-bearing Kuanti Formation in Qujing, Yunnan Province. *Vertebr. Palasiat.* **58**, 249–266 (2020).
15. Rong, J. *et al.* Silurian integrative stratigraphy and timescale of China. *Sci. China Earth Sci.* **62**, 89–111 (2019).
16. Zong, R.-W., Liu, Y.-L., Huang, L.-B., Yin, J.-Y. & Gong, Y.-M. Trilobites from the Silurian "Lower Red Beds" of Wuhan, South China: stratigraphic and paleogeographic implications. *Palaeoworld* **31**, 239–248 (2022).
17. McFadden, P. L. A new fold test for palaeomagnetic studies. *Geophys. J. Int.* **103**, 163–169 (1990).
18. Zhu, Y. *et al.* The oldest complete jawed vertebrates from the early Silurian of China. *Nature* **609**, 954–958 (2022).
19. Jing, X., Yang, Z., Tong, Y., Wang, H. & Xu, Y. Identification of multiple magnetizations of the Ediacaran strata in South China. *Geophys. J. Int.* **212**, 54–75 (2018).
20. Han, Z., Yang, Z., Tong, Y. & Jing, X. New paleomagnetic results from Late Ordovician rocks of the Yangtze Block, South China, and their paleogeographic implications. *J.*

Geophys. Res. Solid Earth **120**, 4759–4772 (2015).

21. Huang, B., Piper, J. D. A., Sun, L. & Zhao, Q. New paleomagnetic results for Ordovician and Silurian rocks of the Tarim Block, Northwest China and their paleogeographic implications. *Tectonophysics* **755**, 91–108 (2019).
22. Gallet, Y. & Pavlov, V. Magnetostratigraphy of the Moyero River Section (North-Western Siberia): Constraints On Geomagnetic Reversal Frequency During the Early Palaeozoic. *Geophys. J. Int.* **125**, 95–105 (1996).
23. Torsvik, T. H. *et al.* Ordovician palaeogeography of Siberia and adjacent continents. *J. Geol. Soc.* **152**, 279–287 (1995).
24. Wu, L. *et al.* The amalgamation of Pangea: Paleomagnetic and geological observations revisited. *GSA Bull.* **133**, 625–646 (2021).
25. Torsvik, T. H. *et al.* Phanerozoic polar wander, palaeogeography and dynamics. *Earth-Sci. Rev.* **114**, 325–368 (2012).
